# Economic and behavioral determinants of forced household savings during the COVID-19 pandemic

**DOI:** 10.1007/s10663-022-09563-8

**Published:** 2023-01-09

**Authors:** Jakub Borowski, Krystian Jaworski

**Affiliations:** Collegium of World Economy, Department of Economics II, SGH Warsaw School of Economics, Rakowiecka 24, Warsaw, Poland

**Keywords:** Pandemics, Household savings, Fiscal policy response, Business cycle synchronization, Inflation differentials, D14, D31, E21, E62, H31

## Abstract

This study seeks to identify the determinants of forced household savings in 16 European Union (EU) member states in 2020. We show that the higher the severity of the COVID-19 pandemic in the state, measured by the intensity of government restrictions or the number of COVID-19-related deaths, the higher the level of forced savings. Such savings also increased with gross domestic product per capita and the financial support provided for households and enterprises by the government. Additionally, savings cultures and personality traits that support compliance with pandemic-related restrictions and enhance coping with the hardship of the pandemic had a positive impact on forced savings. Our results show that while common pandemic shock may lead to discrepancies in forced savings in affected countries, their level depends largely on government response in the form of imposed restrictions as well as financial support for households and enterprises. Therefore, strong fiscal support during the pandemic can be likened to sowing the seeds for post-pandemic recovery, as savings accumulated during the pandemic shock may be used to finance the pent-up demand. This, in turn, suggests that fiscal responses during the pandemic may act as a significant driver of post-pandemic business cycle (de)synchronization and inflation differentials among EU member states and, more importantly, euro-area countries.

## Introduction

The outbreak of the COVID-19 pandemic has resulted in a sharp increase in household savings in many countries (International Monetary Fund 2021). This increase partly resulted from higher precautionary savings, which are a form of unconsumed income attributable to income uncertainty caused by large economic shocks, less generous lending conditions, and consumption smoothing (Ercolani et al. 2021). The increase in household savings was also caused by forced savings, which are additional savings generated by households during the pandemic. These savings emerged due to restrictions imposed by governments in order to limit household mobility and the accompanying risk of infection. These restrictions caused a drop in consumption. Meanwhile, gross household savings are the sum of precautionary and forced savings. Therefore, we assume that before the outbreak of pandemic, forced savings equaled zero and gross household savings were equal to precautionary savings. With the onset of the pandemic, forced savings turned positive.

Like precautionary savings, forced savings constitute a flow variable that represents unconsumed income. The higher this flow, the greater the (forced) increase in a household’s stock of accumulated savings over time. This stock can be used to increase a household’s net assets (by enabling it to buy new assets or repay existing debts). Once pandemic restrictions are lifted, the flow of forced savings is driven asymptotically to zero and households may tap into their accumulated assets to finance postponed consumption (i.e., release their pent-up demand). Breaking down gross household savings into precautionary and forced components is relevant to discussions on adjustments to pandemic shocks. This is because precautionary savings may remain elevated for a long period of time and limit post-pandemic recovery, while forced savings may be utilized quickly as they fuel the pent-up demand once restrictions are dropped. In this study, we identify the determinants of forced household savings in 16 European Union (EU) member states in 2020.

Our work is related to two strands of literature. First, a growing body of literature on the economic effects of pandemics points to a large and protracted negative impact on economic activity and social cohesion. Jordà et al. (2022b) show that pandemics are followed by sustained periods of a depressed natural rate of interest. A fall in the equilibrium interest rate results from fewer investment opportunities and a higher propensity to save, possibly due to greater precautionary savings or willingness to rebuild depleted wealth. Ma et al. (2020) estimate the effects of major global disease outbreaks in the 21st century on GDP growth and several other economic variables. They show that GDP growth falls persistently following pandemics, and that this effect is more pronounced in countries with less aggressive first-year fiscal easing. Goolsbee and Syverson (2021) analyze the impact of COVID-19-related government restrictions on consumer behavior in the US. They show that while overall consumer traffic dropped by 60%, restrictions accounted for only 7% points. Their results suggest that individual choices tied to fears of infection have been a major driver of declining consumer demand during the pandemic. Regressions run by Barro et al. (2020) show that during the 1918–1920 Great Influenza Pandemic, the flu-generated decline in private consumption was markedly stronger than the decline in GDP, thus pointing to an increase in the domestic savings rate. Kratena (2022) shows that in a household demand model with heterogeneous agents, the rebound of consumption after enforced saving due to the lockdown is complete after two periods; these are periods covering restrictions and post-lockdown adjustment. She also shows that significant substitution effects between consumption categories are observed. Furceri et al. (2021) focus on the distributional consequences of major pandemics in the past two decades and show that they led to increases in the Gini coefficients and a greater income share amongst higher-income deciles. Krueger et al. (2020) combine a macroeconomic model with an epidemiological model estimated on Swedish health data. They demonstrate that the “Swedish solution,” that is, letting the pandemic play out with little government intervention and thus allowing for the reallocation of consumption toward relatively safe sectors, helps avoid more than two-thirds of the decline in output and consumption. Their results are in line with those of the simulations performed by Eichenbaum et al. (2020). They extend the canonical epidemiology model to show that the recession associated with pandemics is exacerbated by people’s decisions to cut back on consumption and work to reduce the chances of being infected. Ehnts and Paetz (2021) focus on the interplay between fiscal response to the COVID-19 pandemic shock and the economic fallout of the pandemic. They argue that, to avoid the protracted post-pandemic economic slowdown, fiscal policies in the euro area have to be more expansionary and the ECB has to ensure the solvency of national governments. However, given the large asymmetry in the size of pandemic shock, fiscal expansion would run against debt sustainability in some EU countries. Hence, public debt mutualization scheme is needed to avert a new debt crisis and related aftershocks to economic activity (Barbier-Gauchard et al. 2021).

Second, our study contributes to the ongoing discussion on the determinants of household savings. Cross-country studies for developed and developing economies on this topic are limited. However, empirical studies focusing on theoretically underpinned determinants of household savings point to income growth, real interest rates, inflation, life expectancy, old-age dependency ratio, education, average number of children born per woman over a lifetime, household debt, the gap between genders in higher education attainment and employment, giving childbirth at an old age, preferential income tax rates for households with children, households’ non-financial wealth, stock prices, cultural variables (thrift, trust, religiosity), reliance on direct income taxes, and government transfers as factors that influence household savings (Callen and Thimann 1997; Hüfner and Koske 2010; Hunt et al. 2021; Kool and Muysken 2013; Salotti 2010). Moreover, some empirical literature focuses on the effect of large economic shocks on household savings. Mody et al. (2012) show that at least two-fifths of the sharp increase in household saving rates between 2007 and 2009 can be attributed to the precautionary savings motive triggered by the elevated uncertainty from the Great Recession. Aizenman and Noy (2013) study the degree to which negative income shocks in the past lead to higher saving rates for affected households. They find that a greater experience of past crises results in higher household savings. One can expect that the outbreak of the COVID-19 pandemic will give a new impetus for research on the determinants of household savings in times of large economic shocks.

We analyzed the determinants of forced savings in two steps. First, we used a panel model to explain the fluctuations in the gross household saving rate and we interpreted the residuals from that model as forced savings. Second, we constructed a random-effect panel model to explain the evolution of forced household savings as a percentage of disposable income in EU countries. Our results show that pandemic-induced changes in forced savings depend largely on government response in the form of imposed restrictions, as well as financial support for households and enterprises.

The remainder of this paper proceeds as follows. In Sect. 2, we describe the data and econometric methods used. Section 3 presents the results of this study. The last section concludes the paper and outlines avenues for future research.

## Materials and methods

### Data

Our analysis is based on a sample consisting of 16 member states of the European Union (Austria, Belgium, Czechia, Denmark, Finland, France, Germany, Greece, Hungary, Ireland, Italy, Netherlands, Poland, Portugal, Spain, and Sweden). The remaining EU countries were excluded due to missing data regarding the main endogenous variable, that is, the household saving rate. Our dataset spans from 2011 to 2020, and this range is vindicated by the availability of the endogenous variable for all the countries in question.

The household savings rate was obtained from the Eurostat database. It is defined as gross household savings divided by gross disposable income, with the latter adjusted for the change in pension entitlement of households. The main macroeconomic explanatory variables were extracted from Eurostat (population age structure, wealth ratio, disposable income, income distribution, Gini coefficient), the European Central Bank (credit conditions), and the European Commission (structural general government balance and expected unemployment). Statistics regarding the course of the COVID-19 pandemic (number of cases and related deaths as well as the stringency index) were retrieved from the Our World in Data website. There was no need to interpolate missing values in our sample. Using an augmented Dickey-Fuller test, we rejected the null hypothesis that a unit root is present in a time series sample for all variables used in our estimation (i.e., Table 1). This suggests that we are dealing with stationary covariates.

As we believe that behavioral characteristics may have a significant influence on savings rate, we have turned to the European Values Study 2017: Integrated Dataset (EVS 2020). The European Value Study (EVS) and World Value Survey (WVS) are two large-scale, cross-national, and longitudinal survey research programs. This research dataset is one of the most authoritative and widely-used cross-national surveys in the social sciences. At the moment it is the largest non-commercial cross-national empirical time-series investigation of human beliefs and values ever executed. In line with the Memorandum of Understanding, both organizations agreed to cooperate in joint data collection in 2017. EVS and WVS aimed to conduct a major empirical study of the moral and social values underlying European societies. They include a large number of questions in a few thematic categories (perception of life, work, politics, religion, and national identity, among other things), which have been replicated since the early eighties. We used data from the latest wave of the survey, which was conducted between 2017 and 2020. The survey covers all countries in our sample, except Belgium and Ireland. Therefore, in the models based on this dataset, we had to limit our panel to only the 14 remaining countries.

Data on household saving rates were available at a quarterly frequency, so the explanatory variables were transformed to match this frequency. Daily (regarding the course of the COVID-19 pandemic) and monthly (expected unemployment) variables were transformed into quarterly frequencies by averaging. Economic variables available only in yearly frequency were assumed to maintain the same value in all quarters of a given year. Data on income and credit conditions did not require any transformations, as they were readily available at a quarterly frequency. The macroeconomic data were seasonally adjusted by the respective data providers. Data regarding the course of the pandemic were extracted from their raw form.

### Model

The analysis consists of two steps embedded in the panel regression. First, we obtained information on the level of forced savings in particular countries. We did so by following the practices in the recent study of Dossche and Zlatanos (2020). They divided household savings into two parts. The first part is precautionary savings, willingly accumulated by the public, whose evolution can be explained with the help of macroeconomic variables. The second part is forced, or in other words involuntary, savings, attributable to constraints on the consumption of many goods and services during periods of lockdown imposed amidst the COVID-19 pandemic. The authors propose a panel model to explain the fluctuations in the gross household saving rate, and the residuals from that model are interpreted as forced savings in relation to disposable income.

Second, we propose our own panel model specifications to explain the forced savings obtained in the first step. The potential explanatory variables included selected macroeconomic variables and information regarding the course of the COVID-19 pandemic.

## Results

### Calculation of forced savings

Following Dossche and Zlatanos (2020), we propose a model that explains the evolution of the gross household saving rate. Due to the commonality in the data range for all 16 EU countries between 2011 and 2019, we can estimate a balanced panel model. The gross household saving rate is explained by credit conditions, lead of disposable income growth, expected unemployment, and the wealth ratio. The fourth quarter of 2019 is the end of our estimation sample. Even though data for 2020 were available, they were not included as the impact of the COVID-19 pandemic in 2020 would have distorted the estimated coefficients^1^. The detailed results of our model are presented in Table 1.

For the reader’s convenience, we present estimation results both for a random-effects panel model, as well as a fixed-effects one. The respective coefficients and t-statistics for the two methods are quite similar in value. For further calculations in this paper, we use the random-effects specification, as we cannot reject the null hypothesis of Hausman test (at 5% significance level). Such results suggest that both the random and fixed effects estimates are consistent but the fixed effects are less efficient.

**Table 1:** Panel model explaining saving rate (2011–2019 sample).* Source* Own calculations

Varibles	Random effects		Fixed effects	
Credit conditions	−7.941	(−2.874)***	−7.947	(−2.904)***
Lead of disposable income growth	−29.163	(−8.669)***	−29.216	(−8.772)***
Expected unemployment	0.020	(5.326)***	0.019	(5.239)***
Wealth ratio	0.013	(6.226)**	0.012	(5.656)**
Share of population aged 15–19	−0.539	(−2.220)***	−0.615	(−2.524)***
Intercept	10.068	(5.626)***		
Observations	576		576	
Number of countries	16		16	

t-statistics in brackets

***p<0.01, **p<0.05, *p<0.1

Credit conditions are defined as the flow of credit to the domestic private sector as a percentage of GDP. An increase in credit supply is expected to reduce saving rates, since households can more easily borrow to offset negative income shocks and can thus reduce the holdings of precautionary savings. Although a variable constructed in such a way is an imperfect measure of credit conditions, since it also depends on credit demand, it is widely used in other studies (Mody et al. 2012). The main reason for this is the lack of comparable cross-country measures of credit supply. Our estimates confirm the negative relationship between saving rates and credit conditions.

Precautionary savings are also influenced by expected income, which is included in our model as one-quarter ahead of real disposable income growth. As expected, our model suggests that a reduction in expected income growth increases the saving rate (consumption is smoothed out).

The next driver of precautionary savings is household expectations about future unemployment. Each month, harmonized surveys are conducted by the Directorate General for Economic and Financial Affairs (DG ECFIN) for different sectors of the European Union (EU) and applicant countries. One of the questions in the survey concerns the expected change in the number of unemployed people in the given country over the next 12 months. The respondents answer qualitatively—“increase sharply,” “increase slightly,” “remain the same,” “fall slightly,” “fall sharply,” “don’t know.” The individual answers are then compiled into a single composite value (the difference between the shares of positive and negative answers). The higher this number, the larger is the expected increase in the number of unemployed people. Our model indicates that the expected deterioration in the labor market can be linked with a higher precautionary saving rate. We believe this variable also captures the impact of uncertainty on precautionary savings. We have tried including other variables reflecting the uncertainty (e.g., volatility of economic growth) but they turned out to be statistically insignificant.

Moreover, precautionary savings are explained by the wealth ratio (household net financial assets-to-income ratio). This explanatory variable is defined as the ratio of the households’ net financial assets—which refers to all financial assets minus all financial liabilities—at the end of a calendar year, to the gross disposable income earned by households in that year. This variable is lagged by one year to avoid reverse causality from household savings to wealth. In a buffer-stock model, consumers want to hold a certain optimal wealth-to-income level to buffer possible income shocks (Carroll 1997). Thus, the saving rate should increase in response to a negative wealth shock, since consumers try to re-accumulate assets. However, in our model, the relationship was positive and statistically significant. This result is consistent with the models accentuating the positive link between private saving and the depth and sophistication of financial system (with money/GDP ratio as a proxy) which raises the range of saving vehicles (McKinnon 1973; Edwards 1995). Further, households’ net financial assets may be an imperfect measure of total household wealth. For the countries in our sample, a large portion of wealth may also be accumulated in housing, which could explain the discrepancy between the expected sign of the coefficient and the econometric results. Mody et al. (2012) also reported a positive relationship between the wealth ratio and saving rate for non-G7 countries.

Lastly, precautionary savings are influenced by the demographics. According to the life-cycle hypothesis, saving rates change across different population groups (Modigliani and Brumberg 1954). Younger persons and pensioners tend to dissave, whereas working-age people have a higher propensity to save. We have included the share of population aged 15–19 as an explanatory variable to capture these tendencies. As expected, the higher the share of young people in population, the lower the precautionary savings in relation to disposable income.

**Fig. 1:**
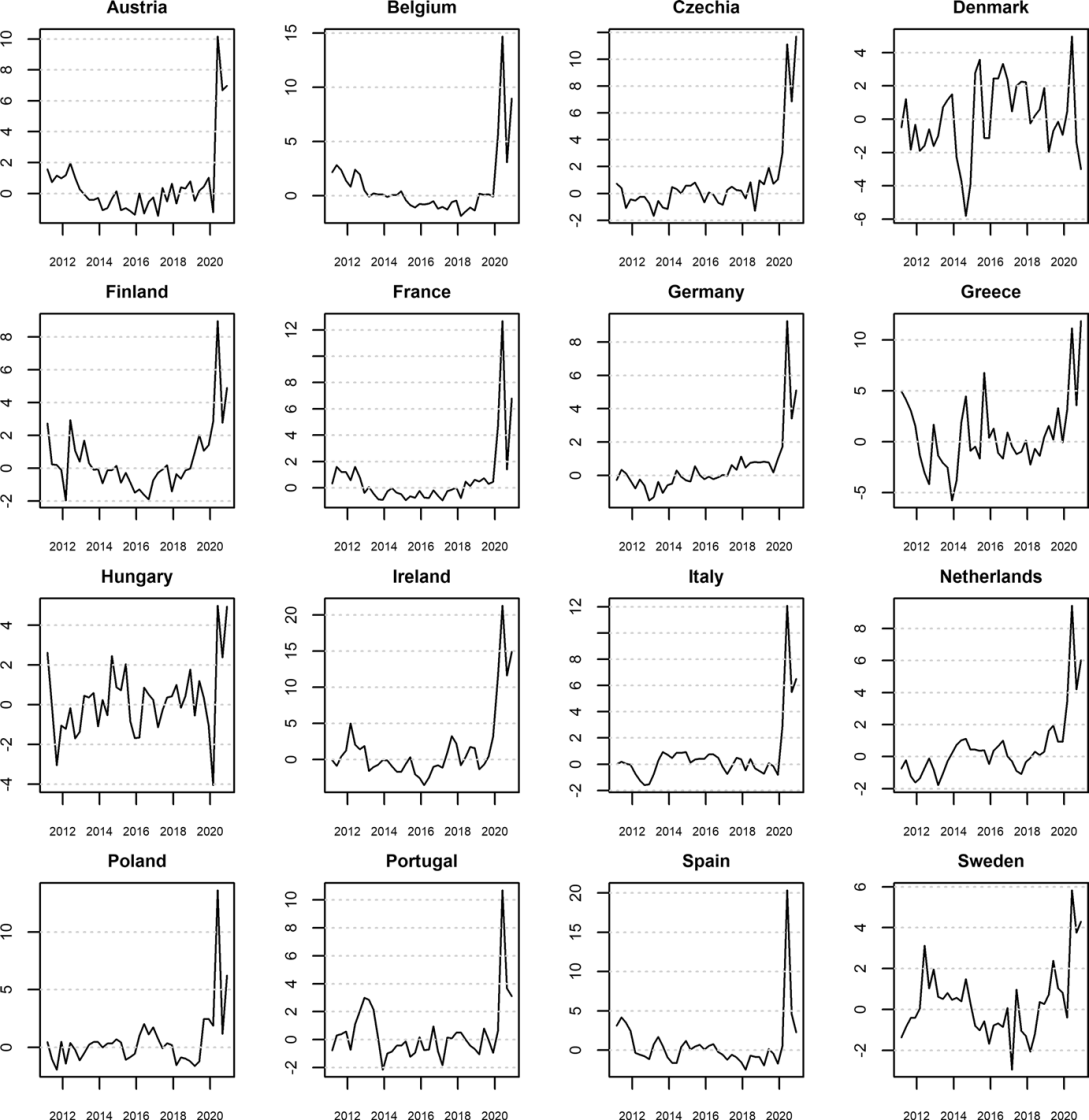
Forced savings as a percentage of household disposable income.* Source* Own calculations

Using the coefficients from Table 1 (random-effects specification), we can calculate the fitted values for the gross saving rate for the years 2011–2020. The difference between actual and fitted gross saving rates is interpreted as forced savings (in relation to disposable income), in line with the methodology of Dossche and Zlatanos (2020). Figure 1 presents these time series. We can see that forced savings hover around zero between 2011 and 2019 in all countries. This means that our model explains the evolution of precautionary savings quite well. Only in 2020, do we see a significant spike in forced savings in relation to disposable income, with the majority of countries seeing double-digit levels of growth. However, in Denmark, Finland, Germany, Hungary, the Netherlands, and Sweden, while the forced savings rate grew rapidly, it peaked below 10% of disposable income. Therefore, one must note that despite the common nature of the pandemic shock, there is significant cross-country heterogeneity regarding forced savings.

### Determinants of forced savings

Once we estimate the level of forced savings as a percentage of disposable income, we can construct a panel model to explain their evolution in 2020. We focus only on the four quarters of 2020, ignoring previous observations. Such an approach is justified as we are interested in determining the determinants of forced savings only during the COVID-19 pandemic. The World Health Organization declared the outbreak of COVID-19 a Public Health Emergency of International Concern on January 30, 2020, and a pandemic on March 11, 2020.

An alternative approach would have been to extend the estimation sample of the model presented in Table 1 to include the year 2020 and add explanatory variables that would capture the evolution of the COVID-19 pandemic. By doing so, we could determine the extent to which pandemic-related covariates were responsible for an increase in the overall household saving rate, which could then be interpreted as forced savings. However, such an approach would prevent us from directly analyzing the economic drivers of forced savings. Any macroeconomic variable included in the specification would be the determinant of both precautionary and forced savings, as their coefficients would reflect their impact on savings not only in 2020 but also in previous years; this would muddy the waters while interpreting the results. Therefore, this method was not used.

In Tables 2 and 3, we present alternative specifications to explain forced savings as a percentage of disposable income. Table 2 focuses on macroeconomic regressors, whereas Table 3 presents the results with regard to behavioral determinants. In each case, we use a random-effects model, as the majority of models include an explanatory variable that was originally available only in yearly frequency, which produces a time-invariant covariate after a conversion into quarterly frequency (we are only using one year—2020—for estimation in Tables 2 and 3). Such characteristics rule out using a fixed-effects model. The aforementioned properties of explanatory variables prevent us also from including more than one macroeconomic or behavioral indicator in each specification as they would be perfectly correlated. Models 1–7 are estimated using the sample of all 16 countries, and models 8–14 are calculated using data from 14 countries (due to lack of data regarding behavioral variables, see Sect. 2.1).

All 14 models included a statistically significant variable representing the evolution of the pandemic, based on either the stringency index or the number of new COVID-19 deaths per million people. We used the government stringency index calculated by the Oxford Coronavirus Government Response Tracker team. It records the strictness of lockdown-style policies that primarily restrict people’s behavior. It is a composite measure based on nine response indicators, including school closures, workplace closures, and travel bans, rescaled to a value from 0 to 100 (100 being the strictest). According to each of the 14 specifications, a higher severity of pandemic (either measured by the intensity of government restrictions or number of deaths) leads to higher forced savings. This result is quite intuitive, as lockdown measures imposed to contain the virus prohibited households from consuming a large share of their normal expenditure basket.

**Table 2:** Macroeconomic models explaining forced savings as a percentage of disposable income.* Source* Own calculations

Variables	Model						
	(1)	(2)	(3)	(4)	(5)	(6)	(7)
Stringency index	0.133 (6.774)***		0.133 (6.784)***		0.133 (6.797)***		0.133 (6.798)***
New COVID-19 deaths per million people		0.007 (3.436)***		0.008 (3.585)***		0.007 (3.442)***	
Change of structural GG balance			−0.802 (−1.729)*	−0.911 (−2.018)**			
Share of population aged 35–39					2.654 (2.554)**	2.613 (2.483)**	
GDP per capita PPP							0.047 (1.931)*
Constant	−1.713 (−1.234)	4.562 (5.061)***	−4.063 (−2.114)**	1.842 (1.161)	−19.441 (−2.752)***	−12.863 (−1.821)*	−6.794 (−2.298)**
R-squared	0.43	0.16	0.45	0.21	0.46	0.23	0.45
Observations	64	64	64	64	64	64	64
Number of countries	16	16	16	16	16	16	16

t-statistics in brackets

***p<0.01, **p<0.05, *p<0.1

We also experimented with the use of the number of new COVID-19 cases as a measure of the severity of the pandemic, but this turned out to be statistically insignificant across all specifications. This is an important result concerning the evolution of forced savings during the subsequent waves of the pandemic. The number of cases may periodically increase again, but thanks to ongoing vaccination, the number of hospitalizations and deaths due to COVID-19 related issues should stay relatively low compared to the first wave of the pandemic observed in 2020. If such a scenario materializes, then forced savings will not spike in the future as violently as they did previously.

### Macroeconomic determinants of forced savings

Models 3 and 4 included the change in structural general government balance (in relation to GDP), between 2019 and 2020, as explanatory variables. This indicator should capture the scale of government support aimed at minimizing the negative impact of the pandemic on economic activity. The measures varied across countries but generally included direct transfers to households and entrepreneurs, credit guarantees, wage subsidies for employees, loans, and additional public spending in the health sector. Our models indicate that the decrease in structural general government balance (fiscal easing) in relation to GDP by 1% point between 2019 and 2020 leads to an increase in forced savings by 0.80–0.91% of disposable income. The most straightforward interpretation of this result is that additional income, in the form of government transfers, is usually not spent, which leads to a build-up of (forced) savings.

The negative sign of the coefficient regarding the change of structural general government balance may also have another interpretation. Furceri et al. (2021) showed, using previous episodes of other pandemics that if fiscal response is strongly supportive, inequality barely increases in the aftermath of pandemics. On the other hand, austerity leads to a significant increase in inequality. If we connect these findings with the study of Davenport et al. (2020), which showed that during the COVID-19 pandemic in the UK only the higher-income households managed to accumulate savings, while lower-income households de-cumulated, we can conclude that fiscal response supported (forced) savings by minimizing income inequalities.

The latter interpretation is also supported by the results of Models 5 and 6. In these models, we included the share of the population aged 35–39 as an explanatory variable. The larger the share, the higher the savings rate in 2020. Such age groups usually consist of well-off individuals with stable employment, who in line with the aforementioned studies were the most likely to accumulate savings during the COVID-19 pandemic. Other specifications showed the 35–44 age bracket to be statistically significant, which also supports our interpretation.

However, we must admit that we estimated models that explicitly used some measure of income inequality (the Gini coefficient or income quintile share ratio). In every specification, this variable turned out to be statistically insignificant. Generally, income inequality does not vary much across the 16 European countries in our analysis (compared to some low-income countries outside the EU). This may explain why the aforementioned variables were not a statistically significant factor in the level of forced savings between countries, even though anecdotal evidence suggests that inequality may impact forced savings due to negative savings in lower-income households. Government financial support during the pandemic may have disrupted this relationship. We view the relationship between income inequality and forced savings as an interesting issue for future research.

Model 7 shows that the coefficient for GDP per capita (PPP based), expressed as a percentage of the EU average, is positive and statistically significant. This means that economic development is an important determinant of forced savings. This result is consistent with theoretical and empirical findings pointing to the nexus between the structure of consumer demand and income level (Buera and Kaboski 2012; Foellmi and Zweimüller 2008). As households with higher incomes tend to spend a greater share of it on services, their forced savings resulting from pandemic-related restrictions are high compared to low-income households.

### Behavioral determinants of forced savings

In Models 8–14, we tested the importance of behavioral factors (combined with the measure of the severity of the pandemic) in forced savings. The results are shown in Table 3. We have used the individual responses from the questionnaire “Social values, attitudes & stereotypes” in the EVS/WVS study (see the Sect. 2.1 for more details). Firstly, we discarded questions for which a connection to forced savings could not be easily established (e.g., statements such as “I agree that a university education is more important for a boy than for a girl”). After such initial screening, the question that captured the link between social values and savings the best was the inquiry about “a list of qualities that children can be encouraged to learn at home.” The respondents were asked to choose up to five qualities that they “considered to be especially important.” Further testing revealed that indicators constructed based on the questions from the EVS/WVS study were statistically insignificant.

**Table 3:** Behavioral models explaining forced savings as a percentage of disposable income.* Source* Own calculations

Variables	Model						
	(8)	(9)	(10)	(11)	(12)	(13)	(14)
Stringency index	0.135 (6.544)***	0.132 (6.334)***		0.137 (6.613)***		0.137 (6.622)***	
New COVID-19 deaths per million people			0.006 (2.697)***		0.007 (2.953)***		0.007 (3.234)***
Thrift, saving money and things	9.842 (1.932)*						
Obedience		15.655 (3.139)***	13.562 (2.197)**				
Good manners				12.907 (1.769)*			
Independence					−5.809 (−1.706)*		
Determination, perseverance						11.718 (2.498)**	11.222 (2.203)**
Constant	−5.754 (−2.713)***	−5.838 (−3.486)***	1.097 (0.751)	−12.738 (−2.155)**	7.159 (3.552)***	−7.268 (−3.158)***	−0.527 (−0.247)
R-squared	0.46	0.49	0.21	0.46	0.18	0.48	0.21
Observations	56	56	56	56	56	56	56
Number of countries	14	14	14	14	14	14	14

t-statistics in brackets

***p<0.01, **p<0.05, *p<0.1

We calculated the share of respondents (after the removal of missing or not applicable answers) that mentioned a particular “important child quality.”The individual answers were multiplied using weights provided in the EVS/WVS study aimed at adjusting some sociodemographic characteristics in the sample population to the distribution of the target population. These weights were computed using the marginal distributions of age, sex, education, and region. For more details, see the EVS/WVS variables report (EVS/WVS 2021).

In Model 8, the share of respondents mentioning “thrift, saving money, and things” turned out to have a statistically significant positive impact on forced savings in relation to disposable income. These results are in line with the study of Kool and Muysken (2013), who found that three cultural variables (thrift, trust, and religiosity), apart from standard macroeconomic variables, explain cross-country saving heterogeneity for the 30 OECD countries during 1990–2010.

Models 9–11 indicate “obedience” and “good manners” as child qualities positively impacting the forced savings. We interpret these results based on citizens’ willingness to comply with government restrictions. The greater the conformity, the greater the effectiveness of government-imposed measures in limiting household mobility and consumer demand, thus leading to higher forced savings. This line of thought is confirmed by the results of model 12, which points to “independence” as a trait that negatively impacts the forced savings. The need for government guidelines limits the accumulation of forced savings.

Finally, the important child qualities of “determination” and “perseverance” positively impacted forced savings. We believe that such characteristics may be responsible for coping successfully with the hardship of the pandemic and managing to get by. Individuals possessing such traits were likely to maintain cash flow during the COVID-19 pandemic, leading to positive forced savings.

## Conclusion

We show that the shock caused by the COVID-19 pandemic has caused large discrepancies in forced savings in EU member states. Our estimates suggest that the volatility of forced savings depends largely on the intensity of government-imposed restrictions or the number of deaths involving COVID-19. Forced savings are also positively affected by GDP per capita and financial support for households and enterprises provided by the government. A strong fiscal response to a pandemic shock usually improves the financial situation of low-income earners, thus allowing higher forced savings. We estimate that greater government support during pandemics proxied by deterioration of structural general government balance in relation to GDP by 1% point led to an increase in forced savings by 0.80–0.91% of disposable income. Furthermore, behavioral factors matter. A culture of saving, personality traits that support compliance with pandemic-related restrictions and help in coping with the hardship of the pandemic have a positive impact on forced savings.

Our results have three policy implications. First, they show that government-imposed restrictions and strong fiscal support will aid in the post-pandemic recovery, as savings accumulated during a pandemic shock may be used to finance the pent-up demand. This conclusion is corroborated by de Soyres et al. (2022) who find that governments that provided generous fiscal support mitigated the drop in goods consumption during lockdowns and boosted consumption in periods of increased household mobility. This means that fiscal packages introduced during the pandemic can be seen as a signal of reduced uncertainty regarding the evolution of future aggregate demand (boosting confidence) and lower probability of protracted economic slowdown (Deb et al. 2021). Nevertheless, the growth effects of forced savings as a source of pent-up demand depend on the way financial assistance is distributed among households. Fiscal packages focusing on low-income earners who have a relatively high marginal propensity to consume are more likely to effectively boost pent-up demand than schemes that lead to the accumulation of assets among households in the upper tail of the income distribution and, in turn, higher income and wealth inequality. These findings offer yet another reason (apart from the one related to social cohesion) for implementing fiscal packages during pandemics in ways that stabilize the income of low earners. Indeed, this was the dominant approach of EU countries in 2020 that overwhelmingly focused their fiscal responses on employment “hibernation” and introduced wage subsidies and other incentives for firms to maintain jobs despite reduced demand and elevated uncertainty.^2^ Almeida et al. (2021) show that, with some exceptions, EU Member States’ discretionary fiscal policy measures taken during the first phase of the pandemic in 2020 proved their worth in limiting increases in poverty and inequality at the country level.

Second, containment measures and fiscal responses during the pandemic may act as a significant driver of post-pandemic business cycle (de)synchronization and inflation differentials among EU member states and, more importantly, euro area countries. The empirical findings for the US suggest that fiscal support measures designed to counteract the severity of the pandemic’s economic effects contributed massively to the large increase in US inflation by the end of 2021 (Jordà et al. 2022a).

Finally, large discrepancies in forced savings are yet another argument for fiscal policy coordination in the euro area. This argument is particularly relevant in the context of longer-lasting pandemics that may require protracted fiscal easing. We view these three issues as promising avenues for future research.

## Data Availability

The data that support the findings of this study are available from the corresponding author upon reasonable request.

## Footnotes

1 As a robustness check, we estimate the model outlined in Table 1 on a sample covering the years 2011–2020 instead of 2011–2019. This modification yields different coefficients and different estimates of forced savings, as we allow the data from the pandemic year (2020) to influence the coefficients. Nevertheless, the forced savings remain positive (cf. Fig. 1) and all determinants outlined in Tables 2 and 3 remain statistically significant.

2 According to Eurostat, the unemployment rate in the EU only moderately increased in 2020 and resumed a downward trend in 2021. Income inequality, measured by the Gini coefficient of equivalized disposable income, dropped in 2020 in comparison with 2019 in 16 EU countries and increased significantly (more than 1% point) only in Malta and the Netherlands.

## References

[CR1] Aizenman J, Noy I (2013) Public and private saving and the long shadow of macroeconomic shocks. NBER Working Paper 19067, National Bureau of Economic Research

[CR2] Almeida V, Barrios S, Christl M, De Poli S, van der Tumino A (2021). The impact of COVID-19 on households income in the EU. J Econ Inequal.

[CR3] Barbier-Gauchard A, Dai M, Mainguy C, Saadaoui J, Sidiropoulos M, Terraz I, Trabelsi J (2021). Towards a more resilient European Union after the COVID-19 crisis. Eurasian Econ Rev.

[CR4] Barro RJ, Ursúa JF, Weng J (2020) The coronavirus and the great influenza pandemic: Lessons from the “Spanish flu” for the coronavirus’s potential effects on mortality and economic activity. NBER Working Paper 26866, National Bureau of Economic Research

[CR5] Buera FJ, Kaboski JP (2012). The rise of the service economy. Am Econ Rev.

[CR6] Callen MT, Thimann MC (1997) Empirical determinants of household saving: evidence from OECD countries. International Monetary Fund

[CR7] Carroll CD (1997). Buffer-stock saving and the life cycle/permanent income hypothesis. Q J Econ.

[CR8] Davenport A, Joyce R, Rasul I, Waters T (2020) Spending and saving during the COVID-19 crisis: evidence from bank account data. Briefing Note, p. 308, Institute for Fiscal Studies

[CR9] de Soyres F, Santacreu AM, Young H (2022) Fiscal policy and excess inflation during Covid-19: a cross-country view. FEDS Notes. Board of Governors of the Federal Reserve System. https://www.federalreserve.gov/econres/notes/feds-notes/fiscal-policy-and-excess-inflation-during-covid-19-a-cross-country-view-20220715.html, accessed 16 September 2022

[CR10] Deb P, Furceri D, Ostry JD, Tawk N, Yang N (2021) The effects of fiscal measures during COVID-19. IMF Working Paper 21/262, International Monetary Fund

[CR11] Dossche M, Zlatanos S (2020) COVID-19 and the increase in household savings: precautionary or forced?. Economic Bulletin Boxes vol. 6, European Central Bank

[CR12] Edwards S (1995) Why are saving rates so different across countries?: An international comparative analysis. NBER Working Paper 5097, National Bureau of Economic Research

[CR13] Eichenbaum MS, Rebelo S, Trabandt M (2020) The macroeconomics of epidemics. NBER Working Paper 26882, National Bureau of Economic Research

[CR14] Ehnts D, Paetz M (2021). COVID-19 and its economic consequences for the Euro Area. Eurasian Economic Review.

[CR15] Ercolani V, Guglielminetti E, Rondinelli C(2021) Fears for the future: saving dynamics after the COVID-19 outbreak. Covid-19 note. https://www.bancaditalia.it/pubblicazioni/note-covid-19/2021/Note_saving_EGR_14giugno2021.pdf., Bank of Italy, accessed 15 April 2022

[CR16] EVS/WVS (2021). European values study and world values survey: joint EVS/WVS 2017–2021 dataset – variable report (documentation/tables). Version: July 2021 2. GESIS Data Archive.

[CR17] EVS (2020) European Values Study 2017: Integrated Dataset (EVS 2017). Data, G. Archive, cologne. ZA7500 Data file version 4.0.0. doi:10.4232/1.13560

[CR18] Foellmi R, Zweimüller J (2008). Structural change, Engel’s consumption cycles and Kaldor’s facts of economic growth. J Monet Econ.

[CR19] Furceri D, Loungani P, Ostry JD, Pizzuto P (2021). The rise in inequality after pandemics: can fiscal support play a mitigating role?. Ind Corp Change.

[CR20] Goolsbee A, Syverson C (2021). Fear, lockdown, and diversion: comparing drivers of pandemic economic decline 2020. J Public Econ.

[CR21] Hunt E, Jeon H, Lee S (2021). Determinants of household savings: an empirical evidence from the OECD member countries. Bus Economic Res.

[CR22] Hüfner F, Koske I(2010) Explaining household saving rates in G7 countries: Implications for Germany. OECD Economics Department Working Papers 754, OECD

[CR23] International Monetary Fund (2021) World Economic Outlook, October. https://www.imf.org/-/media/Files/Publications/WEO/2021/October/English/text.ashx, accessed 15 April 2022

[CR24] Jordà Ò, Liu C, Nechio F, Rivera-Reyes F (2022a) Why is US inflation higher than in other countries? FRBSF Economic Letter 2022(7). Federal Reserve Bank of San Francisco

[CR25] Jordà Ò, Singh SR, Taylor AM (2022). Longer-run economic consequences of pandemics. Rev Econ Stat.

[CR26] Kool CJM, Muysken J (2013). Cross-country private saving heterogeneity and culture. De Economist.

[CR27] Kratena K (2022). Supply constraints in a heterogenous agents household demand model: a method for assessing the direct impact of the COVID lockdown. Empirica.

[CR28] Krueger D, Uhlig H, Xie T(2020) Macroeconomic dynamics and reallocation in an epidemic: evaluating the “Swedish solution”. NBER Working Paper 27047, National Bureau of Economic Research

[CR29] Ma C, Rogers JH, Zhou S (2020). Global economic and financial effects of 21st century pandemics and epidemics. Covid Econ.

[CR30] Mckinnon R (1973). Money and capital in economic development.

[CR31] Modigliani F, Brumberg R, Kurihara K (1954). Utility Analysis and the consumption function: an interpretation of Cross-Section Data. Post keynesian economics.

[CR32] Mody A, Ohnsorge F, Sandri D (2012). Precautionary savings in the great recession. IMF Econ Rev.

[CR33] Salotti S (2010). Global imbalances and household savings: the role of wealth. Soc Sci J.

